# The Influence of Vowels on the Identification of Spoken Disyllabic Words in the Malayalam Language for Individuals with Hearing Loss

**DOI:** 10.3390/diagnostics14232707

**Published:** 2024-11-30

**Authors:** Vijaya Kumar Narne, Dhanya Mohan, M. Badariya, Sruthi Das Avileri, Saransh Jain, Sunil Kumar Ravi, Yerraguntla Krishna, Reesha Oovattil Hussain, Abdulaziz Almudhi

**Affiliations:** 1 Department of Medical Rehabilitation Sciences, College of Applied Medical Sciences, King Khalid University, Abha 61481, Saudi Arabia; sravi@kku.edu.sa (S.K.R.); ksuryanarayana@kku.edu.sa (Y.K.); rehussain@kku.edu.sa (R.O.H.); almudhi@kku.edu.sa (A.A.); 2 Speech-Language Pathology Unit, College of Applied Medical Sciences, King Khalid University, Abha 61481, Saudi Arabia; 3 Department of Speech Pathology and Audiology, Amrutha Institute of Medical Sciences, Kochi 682041, Kerala, India; dhanyam@aims.amrita.edu (D.M.); sruthi.avileri@gmail.com (S.D.A.); 4 Department of Audiology, AWH Special College, Kozhikode 673003, Kerala, India; badariyam@gmail.com; 5 Department of Audiology/POCD, All India Institute of Speech and Hearing, Mysuru 570006, Karnataka, India; saranshjain@aiishmysore.in

**Keywords:** speech identification score, degree of hearing loss, disyllabic wordlists, vowel combinations

## Abstract

Background/Objectives: The present study investigates the reasons for better recognition of disyllabic words in Malayalam among individuals with hearing loss. This research was conducted in three experiments. Experiment 1 measured the psychometric properties (slope, intercept, and maximum scores) of disyllabic wordlists. Experiment 2 examined PB_max_ scores across varying degrees of sensorineural hearing loss (SNHL) and compared these findings with studies in other Indian and global languages. Experiment 3 analyzed the recognition performance of different vowel combinations across varying degrees of hearing loss. Methods: Experiment 1: Psychometric functions for disyllabic word recognition were derived from 45 individuals with normal hearing. Word recognition was tested in quiet at nine hearing levels ranging from −10 to +40 dB HL. Experiment 2: 1000 participants with SNHL were categorized by hearing loss severity (mild, moderate, moderately severe, severe, and profound). Word recognition scores, including PB_max_, were analyzed and compared across severity levels. Experiment 3: Percent error scores for 17 vowel combinations were assessed in 37 participants with SNHL. Ten disyllabic words represented each combination. Results: Disyllabic wordlists showed significantly higher word recognition scores than monosyllabic lists across all degrees of hearing loss. Individuals with mild-to-moderately severe SNHL achieved higher PB_max_ scores, with performance declining at severe- and profound-loss levels. The higher recognition of disyllabic words was attributed to contextual cues and low-frequency vowel-based information, particularly benefiting those with residual low-frequency hearing. Error analysis highlighted the influence of specific vowel combinations on word recognition performance. Conclusions: Disyllabic words are easier to recognize than monosyllabic words for individuals with SNHL due to their rich contextual and low-frequency energy cues. Disyllabic wordlists sustain higher recognition scores up to moderately severe hearing loss but show a marked decline with more severe losses. The phonemic balance of wordlists and vowel combinations significantly influences word recognition, emphasizing the importance of these factors in developing wordlists for clinical use.

## 1. Introduction

Speech audiometry uses stimuli like phonemes, words, or sentences to evaluate speech recognition ability in quiet environments. It is a vital tool for assessing the communication abilities of individuals with hearing loss. In addition to verifying pure-tone thresholds [1,2,3], speech audiometry helps identify lesions in the auditory pathway [1,2], evaluate the benefits of hearing aids, and determine cochlear implant candidacy [2,4]. Speech audiometry is a routine component of diagnostic test batteries in audiology clinics across North America, Europe, and Australia. However, speech audiometry is less commonly performed in other parts of the world due to the lack of appropriate materials in local languages.

Malayalam is a Dravidian language spoken by 38 million people in Kerala, a state in southern India, and the union territories of Pondicherry and Lakshadweep. An additional 6 million Malayalam speakers live in the Gulf region and other parts of the world [5]. Speech materials for speech recognition threshold (SRT) and speech identification scores (SIS) in Malayalam were developed by Kacker and Basavaraj [6], but they were limited to phonemic balancing. Psychometric functions to evaluate the equivalence of these lists were not established. Moreover, systematic studies investigating speech identification scores across varying degrees of hearing loss using these materials are lacking.

In Malayalam and other Dravidian languages, word structures are predominantly polysyllabic [7,8,9]. Longer word lengths provide richer contextual cues, which enhance speech recognition scores, particularly in individuals with hearing loss [10,11,12]. Carlo, Wilson, and Villanueva-Reyes [10] evaluated speech identification scores for monosyllabic, disyllabic, and trisyllabic Spanish words at varying presentation levels. Their findings indicated that monosyllabic words achieved maximum scores at 35–40 dB HL, disyllabic words at 15–20 dB HL, and trisyllabic words at 10–15 dB HL. Comparable results were observed in Mandarin for both disyllabic [12] and trisyllabic words [11]. In English, SIS materials have traditionally been designed using monosyllabic words due to their phonetic simplicity. As detailed above, an increase in the number of syllables within a word increases the chance of a word being heard correctly due to contextual cues in both normal and hearing-impaired listeners.

Yathiraj et al. [13] validated disyllabic wordlists in four Indian languages—Kannada, Hindi, Tamil, and Marathi—and found that many individuals with mild-to-severe sensorineural hearing loss (*n* = 345) performed similarly to normal-hearing participants, achieving scores of 95–100% on speech recognition tests in quiet. Similarly, Soh [14] validated the disyllabic wordlist (SC-10) in the Mandarin language developed by Lee and Lee [15]. They measured SIS in normal-hearing participants (*n* = 29) and sensorineural-hearing-loss individuals (*n* = 23) using SC-10. They found that the hearing-impaired participants with a large number of mild-to-severe SNHL scored as well as the normal-hearing participants, achieving a maximum score of 95–100% for speech recognition in quiet. These results are inconsistent with the existing literature for maximum scores of word recognition tests, which are expected to decrease with a greater severity of SNHL for English [16,17] and Danish [18]. 

Rich contextual cues in disyllabic words likely explain the superior performance of individuals with hearing loss across languages [19,20,21,22]. The resulting scores were inflated by contextual cues derived from possible syllable pairings instead of being a true reflection of the discrimination difficulties a person with hearing loss faces. Moulin and Richard [19] explored reasons for the higher PB_max_ scores for disyllabic wordlists in French, finding that contextual cues are stronger for disyllabic words. The frequency of occurrence of words and the educational level of participants influence these contextual cues. Similarly, in the German language Felty [23] also observed that disyllabic words have a stronger influence on contextual cues than monosyllabic words. 

No validation studies were conducted in Malayalam to understand the performance of hearing-impaired listeners. The first two experiments of the present study were to validate the existing wordlists, and, finally, the third experiment of this study documented the possible reasons for the higher PB_max_ scores observed in Indian languages. 

The currently available Malayalam wordlists were developed in the 1990s [6]. These wordlists were only phonemically balanced; the performance of the lists among individuals with hearing loss was not validated. Previous studies in other Indian languages [13] and Mandarin [14] noted that disyllabic words are easier to perceive, and the majority of hearing-impaired listeners with mild-to-severe hearing loss scored above 80% [13,14]. Therefore, it is important to validate the current wordlist in Malayalam. To validate the wordlists, they were evaluated in two ways: (1) the perceptual equivalence of the wordlists in Experiment 1, and (2) the distribution of PB_max_ scores with an increased severity of sensorineural hearing loss in Experiment 2.

## 2. Experiment 1: Determine the Psychometric Characteristics

### 2.1. Methods

- Subjects: Forty-five adults aged 20–40 years (mean age = 32.46 years; S.D. = 3.17 years) with normal hearing participated in this experiment. Each participant underwent a comprehensive audiological evaluation. Otolaryngologic examinations confirmed the absence of abnormalities in all participants. Hearing thresholds were below 16 dB HL across all octave frequencies (250–8000 Hz) for all participants.
- Procedure: There are two phonemically balanced wordlists in Malayalam [6], each containing 25 meaningful disyllabic words (CVCV structure). Each wordlist was recorded, and the root mean square (RMS) levels were adjusted to 60 dB SPL. Two audio tracks were generated (two wordlists). A three-second interval was maintained between words in each audio track. The participants were randomly assigned to nine groups, each consisting of five individuals. Each group was presented with two randomly selected audio tracks, ensuring no track was repeated. The wordlists were played through the personal computer and routed via the calibrated diagnostic audiometer (Piano, Inventis Inc., Padova, Italy) at nine intensity levels (−10, −5, 0, +5, +10, +15, +2, +3, and +40 dB). Participants listened to the stimuli through Sennheiser HDA200 headphones (Sennheiser electronic, GmbH & Co., Wedemark, Germany). The participants listened to the words and repeated them aloud, with their responses audio-recorded for further analysis.

### 2.2. Results

The percentage of correct word recognition was calculated for each wordlist at each presentation level. The percentage of correct word recognition for each list at each presentation level was computed. Regression slopes and intercepts were derived for both lists using sigmoid function(Equation (1)) based on the percentage of correct scores across nine presentation levels. Using the percentage of correct scores at nine presentation levels, regression slopes and intercepts were calculated using non-linear regression (Equation (1)) for both lists. The equation used here was same as that used by Lee and Lee [15].

(1) $$SIS=1-\frac{e^{\left(a+b\times i\right)}}{1+e^{\left(a+b\times i\right)}}\times 100$$

In Equation (1), ‘*SIS*’ is the percentage of correct recognition, ‘*a*’ is the regression intercept, ‘*b*’ is the regression slope, and ‘*i*’ is the intensity level of presentation in dB HL. The percentage of correct word recognition at any specified intensity level can be predictable when using the regression slope, intercept, and intensity level in Equation (1). 

**Figure 1:**
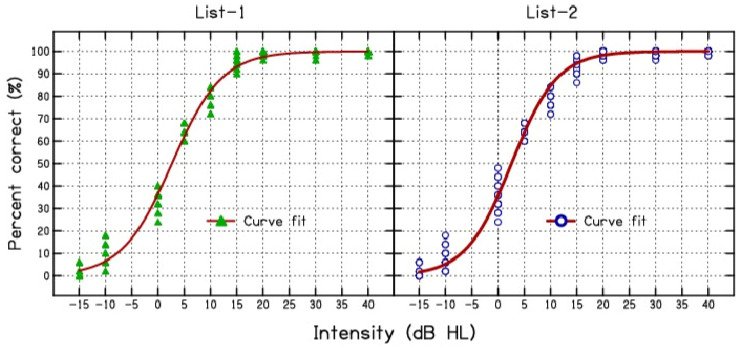
Percentage of correct scores plotted against intensity for both lists in normal-hearing listeners. The solid line represents the fitted psychometric function.

Figure 1 illustrates the curve-fitting results for both list-1 and list-2. The figure indicates that both lists exhibit similar behavior. An independent samples t-test was used to compare the slope and intercept values of the psychometric functions for the two lists. The results revealed no significant differences in the slope (t = −0.65, *p* = 0.5, Cohen’s d = −0.2) or intercept (t = −0.18, *p* = 0.85, Cohen’s d = −0.36) between the two wordlists.

### 2.3. Discussion

The slope, intercept, and levels at which maximum scores were achieved in the present study’s psychometric analysis of disyllabic wordlists closely align with findings from other Indian languages such as Kannada [24], Telugu [25], Tamil [26], and Marathi [27], as well as in other languages like Mandarin [15], Persian [28], and Italian [29]. 

In Persian, the intercept for disyllabic words is approximately 9 dB lower, and the slope is about 5 dB steeper compared to monosyllabic words [28]. Similarly, in the Spanish language, Carlo, Wilson, and Villanueva-Reyes [10] reported that disyllabic word recognition scores reach 100% at 15–18 dB above the threshold, compared to 35–40 dB for monosyllabic words. These studies indicate that disyllabic words are easier to recognize than monosyllabic words. Several factors influence the psychometric properties of disyllabic words across languages and studies. Key factors include the talker’s gender, step size in intensity increments, calibration methods for spoken materials, statistical models used for analysis, and syllable formation [10,23,30]. 

An essential step in validating the current wordlists is determining whether they are psychometrically equivalent and how they compare to similar wordlists in other languages. The results of this study confirm that the current wordlists are psychometrically equivalent and comparable to disyllabic wordlists in other languages. The next step in validation is to evaluate the performance of hearing-impaired participants using these wordlists. 

## 3. Experiment 2: Decrease in PB_max_ Scores with Increased Severity of Sensorineural Hearing Loss

### 3.1. Methods

- Subjects: A total of 1000 participants with varying degrees of hearing loss, referred for hearing aid treatment, were recruited for this study. The subjects’ ages ranged from 17 to 84 years (mean = 52 years, median = 68 years, SD = 20 years). Each participant underwent a comprehensive audiological evaluation. The reports showed that all participants had bilateral sensorineural hearing loss (SNHL) of varying severities and were candidates for hearing aids. Otolaryngological assessments confirmed that no participants required medical or surgical intervention for their hearing loss.
- Procedure:
o
Basic audiological evaluation. All audiological tests were conducted in a double-walled sound-treated room. Pure-tone air conduction thresholds were determined using a calibrated two-channel diagnostic audiometer (Piano, Inventis, Padova, Italy) with TDH-39 headphones (Telephonics, Farmingdale, NY, USA). Bone conduction hearing threshold levels (HTLs) were measured using a B-71 bone vibrator (Radioear, Middelfart, Denmark). HTLs were estimated using the modified Hughson–Westlake procedure. The pure-tone average (PTA) was calculated for each ear separately by averaging the air conduction HTLs at 0.5, 1, 2, and 4 kHz.
o
Speech recognition threshold (SRT). The speech recognition threshold (SRT) in quiet was measured in the same session as pure-tone audiometry using paired words developed by the All-India Institute of Speech and Hearing in Malayalam. Recorded stimuli were presented through the audiometer. The SRT for each ear was determined using the method outlined by ASHA [31].
o
Maximum speech identification score (PB_max_). The PB_max_ score was obtained for each ear using two phonemically balanced Malayalam wordlists [6], as described in Experiment 1. One list was presented for each ear through the audiometer using recorded materials. The speech identification score (SIS) for each ear was estimated using the method outlined by ASHA [31]. Masking in the non-test ear was used when necessary according to standard masking rules [32].
- Statistical Analysis: All statistical analyses were performed using R software version 3.6 [33] and programmed with RStudio [34]. Plots were generated using the ggplot2 package [35].

### 3.2. Results

**Figure 2:**
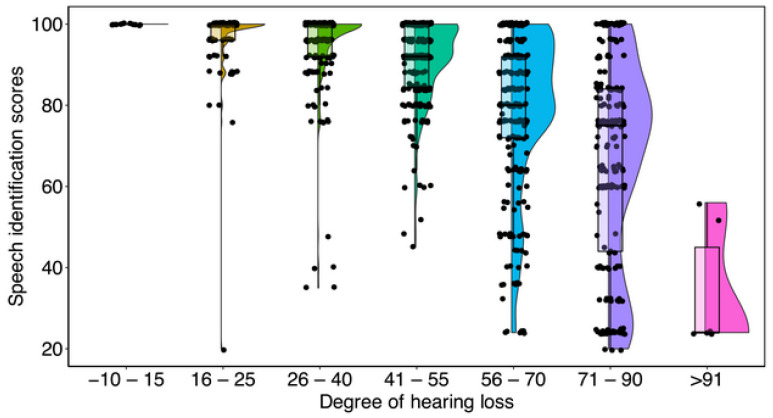
Speech recognition scores as a function of the degree of hearing loss category. A four-frequency PTA categorizes the degree of hearing loss with ranges specified as follows: normal (≤15), slight (16 to 25), mild (26 to 40), moderate (41 to 55), moderately severe (56 to 70), and severe (≥71).

Table 1 presents the mean, median, standard deviation (SD), and range of PB_max_ scores across varying degrees of hearing loss. The data indicate a clear decrease in PB_max_ scores with an increasing severity of hearing loss. Moreover, as PB_max_ scores decline the variability in scores increases, particularly at higher degrees of hearing loss. This trend is visually illustrated in Figure 2, which categorizes PB_max_ scores based on hearing loss severity, as determined by the pure-tone average (PTA) at 0.5 kHz, 1 kHz, 2 kHz, and 4 kHz. A one-way analysis of variance (ANOVA) confirmed a significant reduction in PB_max_ scores with increasing hearing loss severity (F_(5,93)_ = 8.87, *p* < 0.01). Post hoc comparisons with Bonferroni corrections revealed no significant differences in PB_max_ scores for PTAs below 90 dB (*p* = 0.51). However, PTAs exceeding 91 dB differed significantly from all other degrees of hearing loss (*p* < 0.001).

Although PB_max_ scores decrease with increasing hearing loss, it is noteworthy that they remain consistently excellent for individuals with normal hearing to those with moderately severe hearing loss. Specifically, for participants with PTAs in the range of 50-to-85 dB HL, the mean PB_max_ score exceeded 80% in 75% of cases. In contrast, PB_max_ scores showed a sharp decline among individuals with profound hearing loss, as depicted in Table 1 and Figure 2.

**Figure 3:**
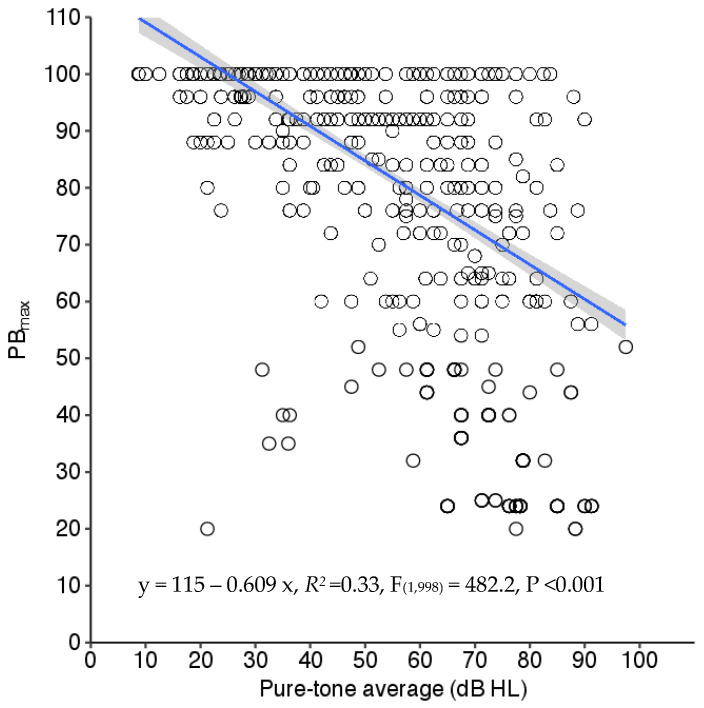
Scatter plot with linear regression shows the relationship between the PB_max_ and pure-tone average (PTA). The shaded area indicates a standard error.

To explore the relationship between the PB_max_ and PTA, a Pearson correlation analysis was conducted, revealing a moderate correlation (r = 0.56, *p* < 0.01) between the two variables. A scatter plot illustrating the relationship, along with a linear regression line, is presented in Figure 3. Linear regression analysis indicated that the PTA accounted for only 30% of the variance in PB_max_ scores. Due to the high variability in PB_max_ scores as a function of the PTA, the correlation alone may not fully capture the complex behavior of PB_max_ across different PTAs. 

### 3.3. Discussion

PB_max_ scores remained high up to moderately severe-to-severe hearing loss, with at least 75% of participants achieving scores above 80% even with severe hearing loss. In contrast, PB_max_ scores deteriorated rapidly for losses greater than severe (Table 1 and Figure 2). However, the range of observed PB_max_ scores increased with the degree of hearing loss. For example, with moderately severe or severe hearing loss, some individuals displayed excellent PB_max_ scores, while others performed poorly. In other words, the range of PB_max_ scores is clustered near excellent when hearing is largely normal and progressively increases in variance with increasing hearing loss. These results align with the studies on disyllabic wordlists in other Indian languages [13], Singapore Mandarin [14], and French [19]. 

However, for monosyllabic words in English, using large-scale studies on PB_max_ scores show that PB_max_ scores start deteriorating from moderate hearing loss [16,36,37,38]. The performance of the hearing-impaired group using the disyllabic wordlist is higher than monosyllabic wordlists across different degrees of hearing loss. One possible reason for the higher performance with disyllabic words may be attributed to the rich contextual cues they provide [19,20,21,22]. 

Moulin and Richard [19] explored the possible reasons for higher scores in disyllabic words across different degrees of hearing loss. They documented that contextual cues and frequency of occurrence play a significant role in a better perception of disyllabic wordlists. Similar observations were made in the German language. In Indian languages, Neha and Narne [39] developed the disyllabic wordlist using a median-to-low frequency of occurrence. As Moulin and Richard [19] documented, the frequency of occurrence has a small effect (10%). Hence, in Indian languages the frequency of occurrence may not contribute significantly. 

In Indian languages, other possible reasons could be due to the dominant low-frequency energy in disyllabic wordlists. Most wordlists in Indian languages take the CVCV form, which has an equal occurrence of vowels [18,24], as vowels have a dominant low-frequency energy. Since most participants with SNHL had better hearing at lower frequencies than at higher ones, it is not surprising that they could guess the words based on vowel or vowel combination information. Thus, the resulting scores were inflated by contextual cues derived from possible syllable pairings instead of being a true reflection of the discrimination difficulties a person with hearing loss faces. The next experiment explores the possible role of vowel pairs in the perception of disyllabic words.

## 4. Experiment 3: Perception of Vowel Combinations in Different Degrees of Hearing Loss

### 4.1. Materials and Methods

**Figure 4:**
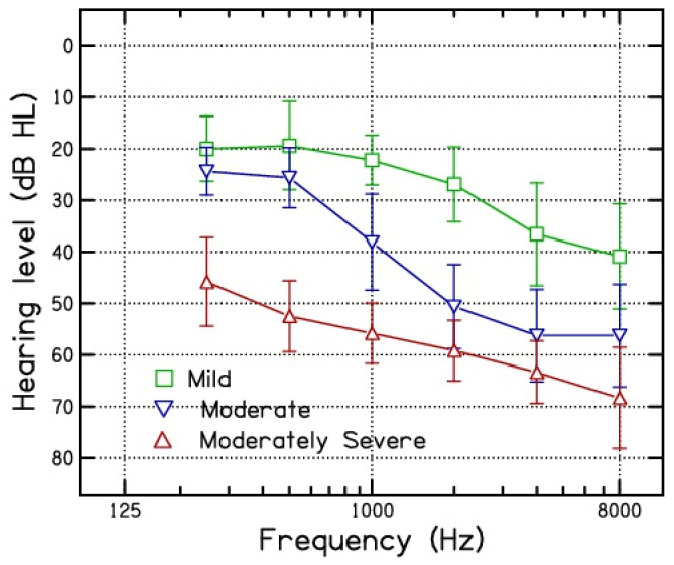
Mean pure-tone thresholds as a function of frequency for three different degrees of hearing loss. The degree of hearing loss is the same as given in Figure 2. The error bar shows the standard deviation.

- Subjects: 37 participants with various degrees of SNHL (mild = 12, moderate = 14, and moderately severe = 11) with flat-to-slightly sloping audiogram configurations were recruited. All participants were experiencing symmetrical hearing loss. The participants’ ages ranged from 25 to 60 years, with a mean age of 34 and a standard deviation of 8.8 years. The participants underwent a complete audiological evaluation, confirming the presence of SNHL at varying degrees, with all participants being candidates for hearing aids. The otolaryngology report confirmed that none of the participants required any medical or surgical treatment for hearing loss. The mean and SD of audiometric thresholds across the frequencies 250 to 8000 Hz of participants are shown in Figure 4.
- Stimuli: Disyllabic words (CVCV) were sourced from the Malayalam dictionary. Malayalam has eleven monophthongs and five diphthongs. From these, 17 vowel pairs (combinations) were selected, which were most commonly occurring in Malayalam (See Table 2). At least ten words for each vowel combination were made with different consonants collected and recorded. A native speaker of Malayalam, a 25-year-old female, spoke these words. The recording was conducted in a quiet environment with a noise level of less than 35 dB SPL. The Rode NT-USB+ (RØDE^©^, Sydney, NSW, Australia) was used to make the recordings. The speaker was instructed to speak at a normal conversational speed and volume. Multiple samples were recorded, and the sample with the minimum perturbation and maximum clarity was selected. Minimum perturbation was based on the Jitter, Shimmer, Harmonic-to-Noise, and Noise-to-Harmonic Ratios, whereas clarity was rated on a seven-point naturalness, clarity, and intelligibility scale, judged by three professional speech–language pathologists. The recordings with minimum perturbation that were decisively judged as the most clear were selected.
- Procedure: The procedures of stimulus presentation and recording the responses for SRT and SIS testing were the same as in Experiment 2.

### 4.2. Results

**Figure 5:**
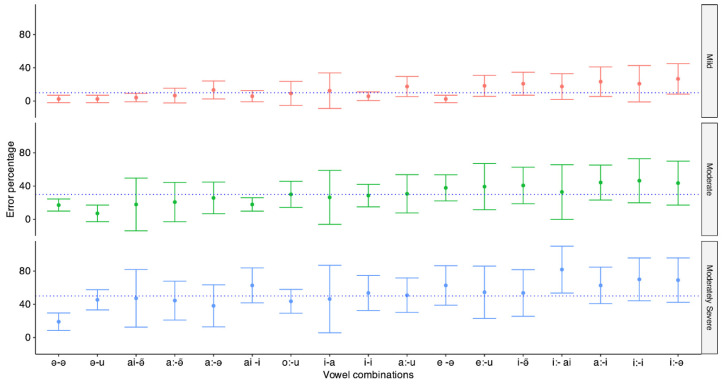
The mean, along with ±1 SD, of the error percentage for disyllabic word identification as a function of vowel combinations for three different degrees of hearing loss. The dotted line indicates the mean error across all vowel combinations for the degree of hearing loss.

Figure 5 illustrates the mean and standard deviation (SD) of the error percentage for each vowel combination across three different degrees of hearing loss. The figure indicates that certain vowel combinations, such as /a - a/, /a - u/, /ai - am/, /a: - am/, and /a: - a/, were relatively easier, showing lower error rates across all degrees of hearing loss. In contrast, vowel combinations including /i/, /a:/, /ai/, and /o/ proved more challenging, with significantly larger error rates and variations observed between different degrees of hearing loss. A mixed ANOVA was performed with the degree of hearing loss as a between-subject factor and the vowel combination as a within-subject factor. The results showed a significant main effect of vowel combinations (F_(16,544)_ = 11.76, *p* < 0.01, ŋP^2^ = 0.13), degree of hearing loss (F_(2,34)_ = 31.46, *p* < 0.01, ŋP^2^ = 0.33), and the interaction between vowel combinations and the degree of hearing loss (F_(32,544)_ = 2.3, *p* < 0.01, ŋP^2^ = 0.45).

To explore the impact of the degree of hearing loss on each vowel combination, a one-way ANOVA was performed for each vowel combination, with Bonferroni correction applied to control for multiple comparisons. The results of the one-way ANOVA are provided in Table 3. Multiple pair-wise comparisons between groups with different degrees of hearing loss are given in Supplementary Materials. These results indicated that error scores were significantly higher for participants with moderately severe hearing loss compared to those with mild hearing loss for most of the vowel combinations.

### 4.3. Discussion

The results showed that the consonants did not exhibit consistent patterns across different degrees of hearing loss. However, vowel combinations showed a systematic pattern across different degrees of hearing loss. Some vowel combinations were easier for all degrees of hearing loss. In contrast, others were more difficult, with the difficulty level increasing as hearing loss worsened. No prior studies have explored the importance of vowel combinations in disyllabic word perception in the hearing-impaired population within the Indian context.

Importance of vowels: Several studies have documented the importance of consonants in word recognition over vowels in normal-hearing listeners for monosyllabic words in the English language [41,42,43]. In contrast, vowels contribute more to the perception of monosyllabic words in the Mandarin language [44]. They attributed that the Mandarin language has more vowels than consonants, and the difference in the ratio of the number of vowels to consonants may partially explain the findings that vowels made a greater contribution to Mandarin [41,42,44]. Like the Madeiran language, the Malayalam language also has a greater frequency of occurrence of vowels (i.e., 60%) compared to consonants (30%) [18]. Furthermore, in the hearing-impaired population and older adults, it is documented that vowels contribute more to monosyllabic word perception [45]. 

In addition, researchers have explored the role of vowel context in word recognition in hearing-impaired populations. For instance, Anderson et al. [46] found that hearing-impaired listeners rely more on vowel cues than normal-hearing listeners, particularly in noisy environments. This aligns with the idea that vowel features, such as formant structure and duration, provide critical perceptual cues for word recognition, especially when consonantal information is degraded due to hearing loss. Similarly, Hedrick et al. [47] emphasized the importance of vowel duration in speech perception among hearing aid users, suggesting that vowel lengthening can enhance intelligibility for individuals with hearing loss. Hence, vowels may be critical in perceiving disyllabic words in Malayalam, at least for the hearing-impaired population. 

Effect of vowel combination: As shown in Figure 4, hearing-impaired participants for disyllabic words having vowels /i/ and /o/ in the final or beginning made more errors compared to disyllabic words having vowels /a/, /a:/, and /u/ in the beginning or final position. There is no such previous research with which we can directly compare the results of the present study. However, some studies have compared the perception of different syllables in different vowel (/a/, /i/, and /u/) contexts. 

The perception of disyllabic words (i.e., CVCV) depends on the correct perception of vowels and consonants. Several studies have explored the influence of vowels on consonant perception in various vowel contexts [48,49]. In English, initial consonants were more accurately identified in syllables containing /a/ followed by /u/, while final consonants were more accurately identified in syllables containing /i/, as reported in previous reports [49,50,51]. Dhanya [40] studied the perception of different consonants (stops, fricatives, and nasals) in three different vowel contexts. They documented that all groups of consonants were better identified in the vowel context of /a/ followed by /u/ and that the lowest levels of identification were in the context of /i/.

Further investigations by Sagi and Svirsky [52] highlighted that vowel-based errors in speech recognition tasks are more common among individuals with cochlear implants, particularly for vowels that share similar formant frequencies, such as /i/ and /e/. These findings point to hearing-impaired individuals’ challenges in distinguishing vowels with close acoustic properties. This may explain why vowel combinations like /i/ and /o/ result in more errors.

Moreover, vowel context has been shown to influence consonant perception in different languages, such as Mandarin and Cantonese, where tonal distinctions further complicate vowel–consonant interactions. According to research by Chen et al. [44], listeners with hearing loss demonstrated a greater difficulty identifying consonants in high-pitched vowel contexts, such as /i/, due to the reduced spectral resolution caused by hearing impairment. This aligns with findings in other non-tonal languages, suggesting that the influence of vowel context on consonant perception is a universal phenomenon across languages.

Given the body of evidence, vowels contribute significantly to the perception of disyllabic words, particularly for hearing-impaired populations. The systematic errors observed with /i/ and /o/ in the current study are consistent with previous research on vowel acoustics and their role in speech perception. Future studies should examine the interaction between vowels and consonants in disyllabic words, especially in languages like Malayalam, where vowel dominance may be critical in overall word intelligibility for hearing-impaired individuals.

## 5. General Discussion

The findings of this study provide significant insights into the psychometric properties of wordlists in Malayalam and the factors influencing their perception across different degrees of hearing loss. The results of the first two experiments indicate that the psychometric properties of the disyllabic wordlists in Malayalam closely resemble those reported for other Indian languages and certain global languages. For instance, the PBmax scores remained excellent (>80%) even in cases of severe hearing loss, a trend consistent with findings in other Indian languages [13] and Italian [19,30]. Despite the robust performance of PB_max_ scores, there is limited research on the underlying factors contributing to this trend in moderately severe hearing loss. Studies in European languages have suggested that contextual cues and frequency of occurrence significantly impact the perception of disyllabic wordlists [19]. Neha and Narne [39] explored the role of frequency of occurrence in Kannada and found that median-to-low-frequency words did not substantially affect scores across different degrees of hearing loss. This suggests that frequency of occurrence may not be the primary factor influencing word recognition in disyllabic wordlists in Indian languages. 

A notable characteristic of disyllabic wordlists in Indian languages, including Malayalam, is their adherence to the CVCV structure. While developing these wordlists, care is taken to balance the frequency of occurrence of individual phonemes to match the linguistic characteristics of the respective language. This practice is adapted from procedures used in monosyllabic wordlist development for CVCV structures. However, beyond phoneme balancing, the current findings suggest that internal patterns, such as vowel and consonant combinations within disyllabic words, also significantly influence perception, particularly in populations with hearing loss.

Experiment 3 examined the impact of vowel combinations on the perception of disyllabic words in Malayalam across varying degrees of hearing loss. The results revealed that disyllabic words containing the vowels /i/ and /o/ were more difficult to perceive than those containing /a/, /a:/, and /u/ in either the initial or final position. These findings align with previous research documenting challenges in perceiving vowels with close acoustic properties, such as /i/ and /o/. Interestingly, the distribution of vowel combinations in the current wordlists does not mirror the natural distribution of vowel combinations in spoken Malayalam. For example, the wordlist used for measuring PBmax in Malayalam comprises 40%, 20%, and 13% of words with the vowel combinations /a-a/, /a-u/, and /u-u/, respectively. Most of these combinations correspond to vowels associated with fewer perceptual errors across all degrees of hearing loss. Only 8% of words include challenging vowel combinations, such as those involving /i/ and /o/. Hence, the current vowel combinations in the wordlist may be one of the most important parameters contributing to the higher scores for all degrees of hearing loss. As shown in Table 2, the current disyllabic wordlists do not follow the percentage distribution of vowel combinations in the Malayalam language. 

The disproportionate representation of vowel combinations with lower error rates may explain the consistently higher PB_max_ scores observed across varying degrees of hearing loss. This suggests that the vowel combinations in the wordlist also play a critical role in its overall intelligibility. Therefore, it may be beneficial to consider not only the frequency of phonemes but also the systematic balancing of vowel combinations during the development of wordlists for clinical and research applications in Indian languages. 

## 6. Limitations and Future Directions

This study’s limitations are that it primarily focuses on vowel combinations only and does not account for the role of consonant combinations or phonotactic rules, which could also significantly influence word perception. Furthermore, this study’s findings are specific to the Malayalam language only and may not be generalizable to other Indian languages with different linguistic structures. Further research could explore how consonant combinations and vowel–consonant interactions influence disyllabic word perception. Additionally, studies could investigate whether balancing vowel combinations in wordlists to better reflect the natural distribution in Malayalam would impact performance, especially in hearing-impaired populations. 

## 7. Conclusions

The present study provides valuable insights into the psychometric properties of disyllabic wordlists in individuals with sensorineural hearing loss. It demonstrates that disyllabic words are easier to recognize than monosyllabic words due to their rich contextual cues and dominant low-frequency energy. The results align with findings in other Indian and various global languages, suggesting that disyllabic wordlists maintain higher recognition scores across mild-to-moderately severe hearing loss. However, a marked decline in PB_max_ scores was observed as hearing loss became severe-to-profound, with an increasing variability in performance. The better recognition of disyllabic words until severe hearing loss is likely driven by the vowel-based cues that significantly assist individuals with hearing loss. These findings highlight the importance of considering word type in speech recognition testing, particularly for individuals with hearing loss. 

## Figures and Tables

**Table 1:** Number of subjects, mean, SD, IQR, minimum, and maximum across different degrees of hearing loss.

PTA Range	Number of Subjects	Mean	SD	25%	75%	Min	Max
−10–15	12	1	0.0	100	100	100	100
16–25	95	97.0	14.0	96	100	20	100
26–40	198	95.0	11.0	92	100	35	100
41–55	223	91.0	10.0	85	100	45	100
56–70	241	79.0	19.0	72	92	24	100
71–90	225	66.0	25.0	44	84	20	100
>91	6	34.0	16.0	24	45	24	56

**Table 2:** The frequency of occurrence, percentage of occurrence in the current wordlist, and percentage of occurrence in the Malayalam language for various vowel combinations. This table is based on the findings of Dhanya [40].

	Current Wordlist		In Malayalam Language
Vowel Combinations	Frequency of Occurrence	Percentage of Occurrence	Percentage of Occurrence
\a-a\	12	40	14
\a-u\	6	20	1.4
\u-u\	4	13	0.3
\i-a\	2	7	0.3
\i:-a\	2	7	5.4
\e:-a\	2	7	0.3
\o-a\	1	3	0.3
\ai a\	1	3	0.3

**Table 3:** The one-way ANOVA results show the impact of the overall degree of hearing loss on each vowel combination. η^2^ indicates the eta-squared value of effect size.

Vowels	F	*p*	η^2^
ə-u	72.87	<0.001	0.811
ə-ə	16.74	<0.001	0.496
a:-u	08.59	<0.01	0.336
a:-ə	04.87	0.23	0.223
a:-ə̃	10.44	<0.05	0.381
a:-i	10.84	<0.05	0.389
ai -i	60.90	<0.001	0.782
ai-ə̃	07.43	<0.05	0.304
e -ə	39.68	<0.001	0.700
i-a	03.20	0.9	0.158
i-i	31.32	<0.001	0.648
e:-u	05.97	0.1	0.260
i:-ə	08.91	<0.05	0.344
i:-ai	17.78	<0.001	0.511
i-ə̃	06.62	0.068	0.280
i:-i	15.73	<0.005	0.481
o:-u	11.25	<0.01	0.398

## Data Availability

Data are available from the corresponding authors mentioned in this research paper.

## Supplementary Materials

The following supporting information can be downloaded at: https://www.mdpi.com/article/10.3390/diagnostics14232707/s1, Table S1: Multiple pair-wise comparisons across different degrees of hearing loss for each vowel combination.
